# Topology-enhanced molecular graph representation for anti-breast cancer drug selection

**DOI:** 10.1186/s12859-022-04913-6

**Published:** 2022-09-19

**Authors:** Yue Gao, Songling Chen, Junyi Tong, Xiangling Fu

**Affiliations:** 1 School of Computer Science (National Pilot Software Engineering School), Beijing University of Posts and Telecommunications, Beijing, China; 2 Key Laboratory of Trustworthy Distributed Computing and Service (BUPT), Ministry of Education, Beijing, China; 3 School of Science, Beijing University of Posts and Telecommunications, Beijing, China

**Keywords:** Graph neural network, Breast cancer, Molecular representation, Drug prediction, Bioinformatics, Deep learning, Feature engineering, Decision support system

## Abstract

**Background:**

Breast cancer is currently one of the cancers with a higher mortality rate in the world. The biological research on anti-breast cancer drugs focuses on the activity of estrogen receptors alpha (ER$\alpha$), the pharmacokinetic properties and the safety of the compounds, which, however, is an expensive and time-consuming process. Developments of deep learning bring potential to efficiently facilitate the candidate drug selection against breast cancer.

**Methods:**

In this paper, we propose an Anti-Breast Cancer Drug selection method utilizing Gated Graph Neural Networks (ABCD-GGNN) to topologically enhance the molecular representation of candidate drugs. By constructing atom-level graphs through atomic descriptors for each distinct compound, ABCD-GGNN can topologically learn both the implicit structure and substructure characteristics of a candidate drug and then integrate the representation with explicit discrete molecular descriptors to generate a molecule-level representation. As a result, the representation of ABCD-GGNN can inductively predict the ER$\alpha$, the pharmacokinetic properties and the safety of each candidate drug. Finally, we design a ranking operator whose inputs are the predicted properties so as to statistically select the appropriate drugs against breast cancer.

**Results:**

Extensive experiments conducted on our collected anti-breast cancer candidate drug dataset demonstrate that our proposed method outperform all the other representative methods in the tasks of predicting ER$\alpha$, and the pharmacokinetic properties and safety of the compounds. Extended result analysis demonstrates the efficiency and biological rationality of the operator we design to calculate the candidate drug ranking from the predicted properties.

**Conclusion:**

In this paper, we propose the ABCD-GGNN representation method to efficiently integrate the topological structure and substructure features of the molecules with the discrete molecular descriptors. With a ranking operator applied, the predicted properties efficiently facilitate the candidate drug selection against breast cancer.

**Supplementary Information:**

The online version contains supplementary material available at 10.1186/s12859-022-04913-6.

## Background

Breast cancer is currently one of the most common cancers in the world with a higher fatality rate. According to the related statistics, more than 2 million new cases of breast cancer were diagnosed, where 0.6 million cases died. It accounted for about 15% of all cancer deaths among women worldwide [1]. Meanwhile, drug development is a process with long period and high candidate attrition rate. It was reported that the attrition rate of drug candidates has reached 90% [2]. Therefore, the research on anti-breast cancer drug with the assistance of in-silico tools is an urgent task pending for solutions.

The research on breast cancer is closely related to estrogen receptors [3, 4]. Studies have found that estrogen receptor alpha (ER$\alpha$) is expressed in no more than 10% of normal breast epithelial cells, but about 50%-80% of breast tumor cells; and the experimental results of mice deficient in the ER$\alpha$ gene show that ER$\alpha$ does play a very important role in the development of the breast. At present, anti-hormone therapy is often used in breast cancer patients with ER$\alpha$ expression, which regulates the level of estrogen in the body by regulating the activity of estrogen receptors. Therefore, ER$\alpha$ is considered an important target for the treatment of breast cancer, and compounds that can antagonize the activity of ER$\alpha$ may be candidate drugs for the treatment of breast cancer [5–7].

In order for a compound to be a candidate drug, in addition to having good biological activity, it also needs to have good pharmacokinetic properties and safety in the human body, collectively known as ADMET (Absorption, Distribution, Metabolism, Excretion, Toxicity) properties [8]. Among them, ADME mainly refers to the pharmacokinetic properties of the compound, which describes the law of the concentration of the compound in the organism over time, and T mainly refers to the toxic and side effects that the compound may produce in the human body. No matter how active a compound is, if its ADMET properties are poor, for example, it is difficult to be absorbed by the human body, or the metabolism rate in the body is too fast, or it has some toxicity, then it is still difficult to become a drug, so ADMET properties need to be optimized.

At present, in the field of drug research, regarding time and cost consuming [9, 10], Quantitative Structure-Activity/Property Relationship (QSAR/QSPR) model is one of the most representative in-silico prediction tools to evaluate biological activity and ADMET properties of candidate drug compounds. By leveraging the disease-related targets, e.g. ER$\alpha$, and modeling them as dependent variables, QSAR/QSPR models can predict new compound molecules with better biological activity, physicochemical property, and toxicological responses, and realize preliminary virtual screening for drugs.

With the development of the field of bioinformatics, diverse machine learning based methods have been proposed and applied into QSAR/QSPR modeling for drug property prediction [11–14]. The process can be generally divided into three stages. The first stage is traditional machine learning method represented by linear regression [15], random forest [16], and support vector machine (SVM) [17]. Such representation methods are dependent on hand-craft discrete features from the descriptors and the fingerprints of molecules to model the ADMET properties [18, 19], which is, however, time-consuming and inefficient. The second stage is sequential-based deep learning method represented by CNN [20–23] and LSTM [24]. Such methods can map the structure of compounds into a sequential dimension and aggregate the molecule-level features. Given their remarkable performance improvement compared with traditional machine learning methods, in recent years, sequential-based deep learning methods have been the most popular in-silico methods for QSAR/QSPR modeling. However, existing sequential-based deep learning methods are still based on hand-craft discrete features from the descriptors and the fingerprints of molecules, which means that these methods cannot further reflect the topological characteristics implicit in the molecular structure.

Recently, the popularity of graph neural networks in the bioinformatics community brings potential to further enhance the molecular representation, which is the third stage of QSAR/QSPR modeling [25–30]. Graph neural networks are naturally suitable for modeling topological structure of non-Euclidean data like molecule and can realize global feature extraction from the global structure [31–35]. Currently, a series of graph-based deep learning methods have been proposed for molecular representation and applied for QSAR/QSPR modeling. For example, Duvenaud et al. [36] proposed convolutional networks on graphs to represent molecular fingerprints, which mapped the features of fingerprints into molecular structure via graph convolution operations. In terms of graph based ADMET prediction, Feinberg et al. [37] utilized a modified graph convolutional networks to model ADMET properties at Merck. Montanari et al. [38] demonstrated that graph convolutional neural networks are much more competitive to predict physicochemical ADMET endpoints; Feinberg et al. [39] proposed PotentialNet which applied graph convolution neural networks to conduct multi-task ADMET property prediction.

Although many variants of graph neural networks have been developed for molecular representation and ADMET prediction, limitations of existing methods still exist. First, existing graph-based methods only map the descriptors into a global molecule-level strucrure, which means that they may fail to mine the intrinsic knowledge implicit in the key chemical substructures of the molecules. The significance of biological substructure within a compound is neglected. Second, compared with feature engineering-based machine learning models, GNN models are generally less sensitive to the source of atomic descriptors [40, 41], which means that graph-based methods are less explainable and are not good at representing known explicit knowledge. Therefore, existing graph-based methods fail to integrate the implicit topological knowledge with the explicit discrete descriptor knowledge. Third, most of the existing graph-based methods for ADMET modeling are modified from graph convolution neural network. Such methods follow the transductive learning strategy, which is more computationally expensive and time-consuming compared with inductive learning strategy. Meanwhile, in terms of QSAR/QSPR modeling task, most of the existing methods focus on ADME or ADMET property prediction, while neglect the prediction of biological activity. In addition, to our knowledge, there is still no graph-based QSAR/QSPR model focusing on anti-breast cancer drug selection.

Inspired by the recent progress claimed above, in this paper, we propose the ABCD-GGNN representation method to topologically realize QSAR/QSPR model for ER$\alpha$ and ADMET prediction. ABCD-GGNN can topologically learn both the structure and substructure representations of molecules and deeply integrate them with discrete molecular descriptor representation, which strongly enhances the molecular representation performance and can realize inductive prediction on activity, property, and toxicity. In addition, we design a whole framework of anti-breast cancer drug selection based on ABCD-GGNN with a decision-support setting. With an extra ranking operator applied based on the predicted properties from ABCD-GGNN, selection of candidate drugs against breast cancer can be efficiently facilitated, which may hugely benefit the research on anti-breast cancer drugs. The contributions of this paper are threefold:

- We propose an Anti-Breast Cancer Drug selection method utilizing Gated Graph Neural Networks (ABCD-GGNN), which topologically learns both the implicit structure and substructure characteristics of a candidate drug, and integrates with explicit discrete molecular descriptors to better generate a molecular-level representation. As a result, activity, property, and toxicity of the candidate drugs can all be inductively predicted.
- We design a whole framework of anti-breast cancer drug selection based on ABCD-GGNN to automatically assist researchers with a decision-support setting. To our best knowledge, this is the first work aiming to deal with anti-breast cancer drug development via graph-based deep learning method.
- Extensive experiments conducted on our collected anti-breast cancer candidate drug dataset demonstrate the outstanding performance of our proposed ABCD-GGNN representation method and the rationality of our designed framework for candidate drug selection.

## Methods

**Fig. 1:**
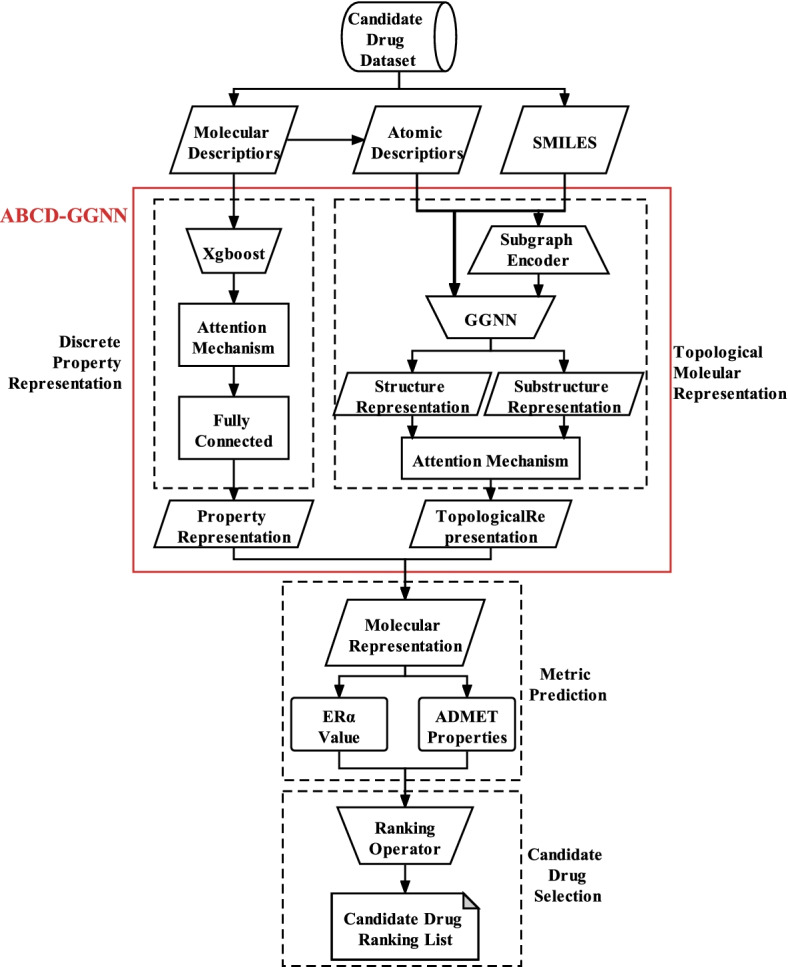
The pipeline of the whole candidate drug selection method

In this section, we first introduce the candidate drug dataset we collect. Then, we illustrate the implementation of our anti-breast cancer drug selection method based on ABCD-GGNN step by step. As the pipeline shown in Fig. 1, our designed drug selection process can be decomposed into four stages: 1) topological molecular graph representation based on GGNN which integrates both structure and substructure characteristics of the molecule, 2) discrete property representation based on machine learning algorithm, 3) integration of the molecular representation of ABCD-GGNN and prediction for ER$\alpha$ and ADMET, and 4) candidate drug selection based on our designed ranking operator.

### Dataset

To evaluate the efficiency of our proposed method, we collect a dataset containing 1974 organic compounds that may be the candidate drugs of anti-breast cancer. The dataset provides the simplified molecular input line entry system (SMILES) and 729 molecular descriptors of each organic compound. The 729 molecular descriptors include diverse descriptions on the characteristics of molecule in two-dimension and three-dimension. The dataset labels the ER$\alpha$ value expressed as pIC50 for each organic compound. Meanwhile, to objectively evaluate the pharmacokinetic properties and the safety of each organic compound, the dataset quantifies them with 5 property labels: absorption, distribution, metabolism, excretion, and toxicity (ADMET). In our collected dataset, the 5 properties are referred to 5 metrics: Caco-2, CYP3A4, human Ether-a-go-go Related Gene (hERG), and Human Oral Bioavailability (HOB), respectively. Due to the page limit of the paper, we present a detailed illustration of a candidate drug sample in Additional file 1: Table 1 of the Appendix section.

### Topological molecular graph representation

**Fig. 2:**
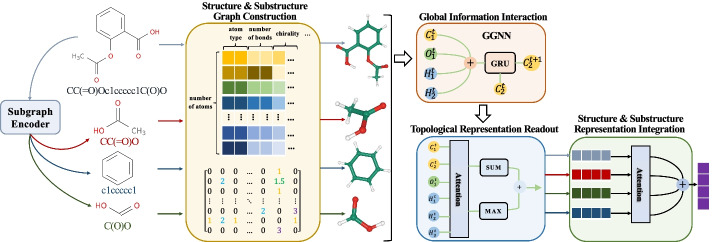
Framework of the topological molecular graph representation for the ABCD-GGNN representation method

In the stage of topological molecular graph representation, graph neural networks are adopted to atomically model the structure of a drug so as to learn the topological molecular features three-dimensionally for the final representation of ABCD-GGNN. With the atom node information globally interacted in the graph structure, both topological structure and substructure features can be well represented and integrated. We first illustrate the implementation of the topological structure representation. Then we illustrate how topological substructure representations are generated and integrate with the topological structure feature to enhance the topological molecular representation. The whole framework of the topological molecular graph representation based on ABCD-GGNN is shown in Fig. 2.

#### Atom-level topological structure graph construction

Graph construction is the kernel stage for the topological graph representation. Given that a graph is denoted as $G=(V,E)$ where $V(|V|=n)$ is the set of graph nodes and *E* is the set of graph edges. In terms of atom-level graph construction for candidate drugs. *V* denotes the atom set in a molecule and *E* denotes the chemical bond set in a molecule.

In terms of the feature initialization for each atom node, here we summarize 8 atomic descriptors from the corresponding SMILES and 729 molecular descriptors, which are atom type, number of bonds, formal charge, chirality, hydrogen bound number, hybridization, aromaticity, and atom mass. The detailed descriptions on the 8 atomic descriptors are listed in Table 1. Every atomic descriptor is transferred into a one-hot vector and are concatenated to form a 39-dimension vector as the initialization of an atom feature.

**Table 1:** Descriptions of components of the feature initialization for the atomic nodes

Atomic descriptor	Description	Vector size
Atom type	12 types of atoms in the 200 molecules of the dataset	12-digit 0/1 vector
Number of bonds	The number of chemical bonds that the atom participates in	6-digit 0/1 vector
Formal charge	The integer-form electric nucleus of the atom	5-digit 0/1 vector
Chirality	CW, CCW, unspecified, or other	4-digit 0/1 vector
Hydrogen bound number	Atomic bound hydrogen atom charge	5-digit 0/1 vector
Hybridization	sp, sp2, sp3, sp3d, or sp3d2	5-digit 0/1 vector
Aromaticity	Whether the atom is part of an aromatic hydrocarbon	1-digit 0/1 vector
Atom mass	The mass of the atom	A normalized number between 0 and 1

In terms of edge construction for each molecular graph, we construct an adjacent matrix $A\in R^{|V|\times |V|}$ to describe the connection relationship between atom nodes. The element in *A*, e.g., $a_{i,j}$ is the connection type between i-th node and node j-th node. The connection type varies among 0, 1, 2, 3, and 1.5, which denotes the bond type: single bond, double bond, triple bond, and aromatic hydrocarbon, respectively.

#### Graph-based global information interaction

Getting the molecular graph constructed, we then employ GGNN [42] to realize the global information interaction between the atom nodes. GGNN learns node representations through neural networks with gated recurrent units (GRU), so that information from neighborhood can be fused and enrich the own representation. Information fusion between nodes strengthens continuously with the interaction time *t* increased and can finally achieve global information interaction of the whole topological structure. In this way, we can finally get a topological structure representation for a candidate drug. Detailed interaction functions are listed as follow:

1 $$\begin{aligned} o^t= & {} \left( W_oA+b_o\right) h^{t-1} \end{aligned}$$

2 $$\begin{aligned} z^t= & {} \sigma \left( W_zo^t+U_zh^{t-1}+b_z\right) \end{aligned}$$

3 $$\begin{aligned} r^t= & {} \sigma \left( W_ro^t+U_rh^{t-1}+b_r\right) \end{aligned}$$

4 $$\begin{aligned} \widetilde{h^t}= & {} tanh\left( W_ho^t+U_h\left( r^t\odot h^{t-1}\right) +b_h\right) \end{aligned}$$

5 $$\begin{aligned} h^t= & {} \widetilde{h^t}\odot z^t+h^{t-1}\odot \left( 1-z^t\right) \end{aligned}$$

where $\sigma$ is the sigmoid function, and parameters *W*, *U*, and *b* are trainable weights and biases. $o^t$ denotes the information that a node could receive from its adjacent neighbors in time step t. $z^t$ and $r^t$ are functions that control update gate and reset gate, respectively, which determine to what degree the neighborhood information contributes to the current node embedding.

#### Topological molecular representation readout

With the topological structure representation of the distinct molecule updated, we then aggregate the atom-level representations into a molecule-level representation in the readout stage. The readout functions are designed as follow:

6 $$\begin{aligned} h_{w,d}= & {} \sigma \left( f_1\left( h_w^t\right) \right) \odot tanh\left( f_2\left( h_w^t\right) \right) \end{aligned}$$

7 $$\begin{aligned} h_G= & {} \frac{1}{\vert W\vert +1} \sum _{w\in W}{h_w + Maxpool\left( h_1,\ldots ,h_w,h_d\right) } \end{aligned}$$

where $f_1$ and $f_2$ are two multilayer perceptrons (MLP) which perform as a soft attention weight and a non-linear feature transformation, respectively.

The readout functions are designed as above with the intention to reflect the truth that all atom node representations contribute to the information aggregation by getting through averaging function and a max-pooling function, while only part of atom nodes with higher weights distributed by attention mechanism contribute more [34]. Consequently, here we get the topological structure representation of the molecule $h_G$ for further prediction.

#### Substructure graph construction and integration

Subgraphs are believed to imply significant attribute characteristics that may further extract and enhance the original graph representation [43], especially to the graph representation of molecules whose substructures represent scaffolds of molecule which should imply much attribute knowledge.

Therefore, we additionally extract the substructures from SMILES of the molecules via the SMILES pair encoding algorithm. Given $G_{sub}={S_1,\cdots ,S_n}$ denotes the subgraph set of n substructures extracted from the graph *G*. Then, we construct the atom-level subgraphs and get through the global interaction via GGNN and representation readout operations as the original graph does. Consequently, we get a substructure-level representation set $H_{sub}={h_{S_1},\cdots ,h_{S_n}}$.

Considering that the contributions different substructures make to the molecular representation are uneven, here we adopt an attention mechanism to dynamically adjust the weights of the original graph and each subgraph. In this way, both molecular graph representation and diverse substructure graph representations get deeply integrated. In other words, the topological graph representations of the candidate drugs are strongly enhanced. Detailed formulas of the attention mechanism and the feature integration is shown below:

8 $$\begin{aligned} w_j= & {} \frac{\exp {\left( e_j\right) }}{\sum _{k\le \vert H_{sub}\vert +1}\exp {\left( e_k\right) }}\ ,\ e_j=c^T\tanh {\left( Wh^j+b\right) } \end{aligned}$$

9 $$\begin{aligned} h= & {} w_0\times h_G+w_1\times h_{S_1}+\cdots +w_n\times h_{S_n} \end{aligned}$$

where ${w_0,\cdots ,w_n}$ is distributed attention weights. *c*, *W*, and *b* are trainable parameters to be learned. Consequently, here we finally get the topological molecular graph representation h that deeply integrate the structure and substructure characteristics of the molecule.

#### Discrete molecular descriptor representation

Molecular descriptors are the discrete expression of a molecule which may imply the potential chemical properties as a candidate drug. Given that the anti-breast cancer candidate drug dataset provides 729 molecular descriptors of all the candidate drugs, which is a quite large number. Here we first employ XGBoost algorithm to select the descriptors that count more. Then, we further reduce the dimensionality of the integrated molecular descriptor representation to realize the molecular descriptor representation readout.

#### Discrete molecular descriptor selection

Considering the redundancy and sparsity of the raw molecular descriptors, we believe it is necessary to select the more property-related descriptors with the help of machine learning method. Therefore, here we apply XGBoost, a decision-tree-based ensemble Machine Learning algorithm that uses a gradient boosting framework, to select the top 50 property-related descriptors for further feature integration and readout.

In terms of the implementation of XGBoost, we first set the objective function, i.e., the loss function as below:

10 $$\begin{aligned} L\left( \phi \right)= & {} \sum _{i} l\left( y_i,\widehat{y_i}\right) +\sum _{k}\Omega \left( f_k\right) \end{aligned}$$

11 $$\begin{aligned} \sum _{k}\Omega \left( f_k\right)= & {} \Upsilon T+\frac{1}{2}\lambda \vert \omega \vert ^2 \end{aligned}$$

where $L\left( \phi \right)$ is the differentiable convex loss function, which represents the gap between the predicted value $\widehat{y_i}$ and the target value $y_i$ to avoid under-fitting; the function $\sum _{k}\Omega \left( f_k\right)$ can reduce the complexity of the model. The additional regularization term helps avoid overfitting. When the regularization parameter is set to 0, the goal is back to the traditional gradient tree boosting algorithm. Since the model is trained by addition, the prediction at time step t equals the prediction at time step t-1 plus the function at time step t. The formula is shown below:

12 $$\begin{aligned} \widehat{y_i^{\left( t\right) }}=\sum _{k}{f_k\left( x_i\right) }=\widehat{y_i^{\left( t-1\right) }}+f_t\left( x_i\right) \end{aligned}$$

Second, we utilize Taylor expansion formula for approximation.

13 $$\begin{aligned} L\left( \phi \right) \approx \sum _{i}\left[ l\left( y_i,\widehat{y_i^{\left( t-1\right) }}\right) +g_if_t\left( x_i\right) +\frac{1}{2}h_if_t^2\left( x_i\right) \right] +\Omega \left( f_t\right) \end{aligned}$$

where $g_i=\partial _{\widehat{y_i}\left( t-1\right) }l\left( y_i,\widehat{y^{\left( t-1\right) }}\right)$ and $h_i=\partial _{\widehat{x_t}\left( -1\right) }^2l\left( y_i,\widehat{y^{\left( t-1\right) }}\right)$ are the first partial derivative and the second partial derivative, respectively.

To make each sample on a leaf node, the node score is defined as $f_t\left( x\right) =W_q\left( x\right)$. The optimal weight is defined as $W_j^*=-\frac{G_j}{H_j+\lambda }$ according to the quadratic function to find the most value formula, where $G_j=\sum _{i} g_i, H_j=\sum _{i} h_i$. Thus, the optimal function value is defined as $obj=-\frac{1}{2}\sum _{j}\frac{G_j^2}{H_j+\lambda }+\gamma T$ and can rank the most properties-related molecular descriptors consequently. Discrete molecular descriptor representation readout With the 50 molecular descriptors selected, we then concatenate them in to a 50-digit vector as a molecule-level representation. Since the contribution of each descriptor, as is ranked by XGBoost algorithm above, should be uneven, we adopt the attention mechanism to dynamically adjust the weight of each digit. Then, to further integrate the discrete molecular descriptor representation so as to better integrate with the topological molecular representation, we reduce the dimensionality of the molecular descriptor representation in to a 39-digit vector with a fully connected layer, which make the two representation readouts in the same size. The formulas are shown below:

14 $$\begin{aligned} w_j= & {} \frac{\exp {\left( e_j\right) }}{\sum _{k\le |m|}\exp {\left( e_k\right) }}\ ,\ e_j=c^T\tanh {\left( Wm_j+b\right) } \end{aligned}$$

15 $$\begin{aligned} m= & {} {[w}_0\times m_0,\ w_1\times m_1,\cdots ,w_n\times m_{|m|}] \end{aligned}$$

16 $$\begin{aligned} h_m= & {} Wm+b \end{aligned}$$

where $h_m$ is the representation readout of the discrete molecular descriptors.

### Metric Prediction

Based on the topological graph representation and the molecular descriptor representation, a final representation of anti-breast candidate drug can be integrated to predict both the ER$\alpha$ value and the ADMET properties.

#### Topological and discrete property representation integration

To adaptively adjust the contribution the topological graph representation and molecular descriptor representation make to the prediction result, we design the hyper parameter $\lambda \in (0,1)$ to weight and integrate the two types of features as the formula shown below:

17 $$\begin{aligned} h_{ABCD-GGNN}=\lambda h+\left( 1-\lambda \right) h_\mathrm {m} \end{aligned}$$

where $h_{ABCD-GGNN}$ is the final integrated representation of the anti-breast candidate drugs.

In this stage, we can claim that the molecular representation based on ABCD-GGNN is completed.

#### Prediction and training process

We treat the prediction of ER$\alpha$ value and ADMET properties as a regression task and a two-class classification task, respectively. In terms of ER$\alpha$ value prediction, the representation $h_{ABCD-GGNN}$ gets fed into a fully connected layer. Parameters are trained through the mean square error.

18 $$\begin{aligned} \widehat{y_{ER\alpha }}= & {} Wh_{ABCD-GGNN}+b \end{aligned}$$

19 $$\begin{aligned} Loss= & {} \frac{1}{m}\sum _{i=1}^{m}\left( y_{ER\alpha }-\widehat{y_{ER\alpha }}\right) ^2 \end{aligned}$$

where *W*, *b* denote trainable parameters, m denotes the batch size, and $y_{ER\alpha }$ denotes the ground truth value of ER$\alpha$.

In terms of ADMET properties prediction, the representation $h_{ABCD-GGNN}$ gets fed into a softmax layer to make prediction. Parameters are trained through the cross-entropy function.

20 $$\begin{aligned} y_{{A\widehat{{DM}}ET}} = & {} softmax\left( Wh_{ABCD-GGNN}+b\right) \end{aligned}$$

21 $$\begin{aligned} Loss= & {} -\sum _{i}{y_{ADMET}log\left( y_{{A\widehat{{DM}}ET}} \right) } \end{aligned}$$

where *W*, *b* denote trainable parameters and $y_{ADMET}$ denotes the i-th element of the one-hot label.

#### Candidate drug selection

To comprehensively consider both the two types of attributes when evaluating the potential of the candidate drugs, here we design a ranking operator consisting of feature binning and scorecard. By scoring each candidate drug, a ranking list can be generated, which can efficiently facilitate the research on anti-breast cancer drug selection.

#### Feature binning

Since the ADMET properties are binary while the ER$\alpha$ value is a continuous value, here we select chi-square binning. The adjacent intervals are the smallest chi-square value are merged together until the definite stopping criterion is met. we set the chi-square threshold (obtained from the significance level and degree of freedom), and calculate the chi-square for each pair of adjacent values as the formula shown below:

22 $$\begin{aligned} x^2=\sum _{i=1}^{2}\sum _{j=1}^{2}\frac{\left( A_{ij}-E_{ij}\right) ^2}{E_{ij}} \end{aligned}$$

where $A_{ij}$ is the feature number of the j-th class attribute in the i-th interval, and $E_{ij}$ is the expectation of $A_{ij}$.

#### Setup of scorecard

To set up the scorecard for the candidate drug ranking, we first calculate the corresponding score of the attribute as below:

23 $$\begin{aligned} \left( \mathrm {\ }{\mathrm {woe\ }}_i*\beta _i+\frac{a}{n}\right) *\mathrm {\ factor\ }+\frac{\mathrm {\ offect\ } }{n} \end{aligned}$$

where$\mathrm {\ }{\mathrm {woe}}_i=\ln {\frac{py_i}{pn_i}}$ is the woe value calculated based on the results of binning and denotes the difference between the response value and the non-response value, $\beta _i$ is the regression coefficient, $\frac{a}{n}$ is the regression intercept term, $\mathrm {factor}$ is the scale factor, and $\frac{\mathrm {\ offect\ }}{n}$ is the offset.

Finally, the calculation formula of the scorecard is defined as below to get the scores of the candidate drugs.

24 $$\begin{aligned} \mathrm {\ score\ }=\sum _{i=1}^{n}\left( \left( \mathrm {\ }{\mathrm {woe\ }}_i*\beta _i+\frac{a}{n}\right) *\mathrm {\ factor\ } +\frac{\mathrm {\ offect\ } }{n}\right) \end{aligned}$$

## Results

In this section, 1) we first evaluate the performance of our proposed ABCD-GGNN on our collected anti-breast cancer candidate drug dataset and compare them with other representative models. 2) Then, we make extensive characteristics analysis and ablation study to demonstrate the effectiveness and contribution each stage makes for the ABCD-GGNN representation. 3) Finally, we demonstrate the biological rationality of applying the ABCD-GGNN prediction results into the ranking operator for candidate drug selection.

### Performance of ABCD-GGNN

#### Baselines and evaluation metrics

Keeping track of the representation methods applied in the study on drug prediction, we compare the representation performance of ABCD-GGNN with those of representative baseline models, which can be categorized into two types: 1) traditional machine learning methods, for example, Linear Regression and Random Forest for ER$\alpha$ prediction and SVM for ADMET prediction; 2) deep learning methods, for example, Bi-LSTM and Graph-CNN for ADMET prediction. Detailed descriptions of these baslines are shown as follow:

- *Linear Regression* a representative supervised learning method. Based on one or more independent variables, linear regression can model a best-fitting relationship for regression problem.
- *Random Forest* an ensemble learning method that constructs decision trees during training. It can realize prediction on the mean prediction of trees for regression tasks by utilizing random subspace method and bagging during tree construction.
- *SVM* a traditional supervised learning method. By maximizing the margin between data samples, SVM can perform well on both regression and classification problem.
- *Bi-LSTM* a representative sequential deep learning method which consists of two LSTMs: one forward and the other backwards direction. Bi-LSTM effectively capture the contextual information in time dimension.
- *Graph-CNN* one of the most representative graph neural network method. By combining CNN with spectral theory, Graph-CNN is more advantageous in dealing with the discriminative feature extraction of signals in the discrete spatial domain and can better describe the intrinsic relationship between different nodes of the graph.

To better reflect the performance of the compared models, in the ER$\alpha$ prediction task, we adopt the of mean square error loss (MSE) and R-Square (R2) as the evaluation metric, while in the ADMET prediction task, we adopt the of mean square error loss (MSE) and R-Square (R2) as the evaluation metric, Precision, Recall, F-score (F1), Area Under the ROC Curve (AUC), and Area Under the Precision-Recall curve (AUPR) as the evaluation metric.

#### Experimental settings

In terms of the detailed dataset setting, we keep the ratio of positive samples and negative samples close to 1:1 for each property. In addition, we utilize ten-fold cross-validation to evaluate the performance of all the compared methods. Positive and negative samples are kept balanced in each fold. We divide the dataset in a ratio of 8:1:1 as training set, validation set, and test set, respectively. The hyperparameters were tuned according to the performance on the validation set. Empirically, we set the learning rate as 0.01 with Adam optimizer and the dropout rate as 0.5. The interaction step of GGNN is set as 2. The hyper parameter $\lambda$ is set as 0.6.

**Table 2:** Performance comparison on the prediction of ER$\alpha$

Model	MSE	R2
Linear Regression	2.156	0.276
Random Forest	0.5147	0.6133
SVM	0.6878	0.6273
ABCD-GGNN	0.4811	0.7741

We run all models 10 times and report the mean test MSE and R2

**Table 3:** Performance comparison on the prediction of ADMET

Model	Dataset	Precision	Recall	F1	AUC	AUPR
SVM	MN	0.7843	0.6709	0.6943	0.7957	0.8209
	HOB	0.7733	0.7498	0.7607	0.8104	0.6239
	hERG	0.8080	0.7589	0.7791	0.8239	0.8494
	CYP3A4	0.8397	0.7998	0.8133	0.8518	0.8591
	Caco-2	0.8453	0.7807	0.8068	0.8552	0.7525
BiLSTM	MN	0.8226	0.7310	0.7537	0.8195	0.7731
	HOB	0.7462	0.7008	0.7165	0.7711	0.7337
	hERG	0.8350	0.7914	0.7968	0.8452	0.8196
	CYP3A4	0.8838	0.8627	0.8741	0.9129	0.8952
	Caco-2	0.8134	0.7954	0.8021	0.8533	0.8258
Graph-CNN	MN	0.8629	0.8293	0.8461	0.8710	0.8623
	HOB	0.8110	0.7635	0.7824	0.8369	0.8061
	hERG	0.8495	0.8690	0.8556	0.9081	0.8585
	CYP3A4	0.8913	0.8827	0.8840	0.9304	0.8731
	Caco-2	0.8479	0.8227	0.8306	0.8740	0.8881
ABCD-GGNN	MN	0.9255	0.9613	0.9430	0.9714	0.9862
	HOB	0.8637	0.8804	0.8712	0.9130	0.9273
	hERG	0.8914	0.8839	0.8842	0.9303	0.9456
	CYP3A4	0.9474	0.9163	0.9355	0.9487	0.9322
	Caco-2	0.8828	0.8832	0.8829	0.9296	0.9134

We run all models 10 times and report the mean test precision, recall, F1, AUC, and AUPR

#### Performance of ABCD-GGNN

The performance of the compared models on the prediction of ER$\alpha$ and ADMET are presented on Tables 2 and 3, respectively. It can be observed that our proposed ABCD-GGNN outperforms all the representative models on the two prediction methods. Specifically, in the ER$\alpha$ prediction task, ABCD-GGNN achieves the lowest loss value and highest R2 value, which means that the prediction results of our proposed model can better fit the expected ER$\alpha$ value with lower error. In the ADMET prediction task, ABCD-GGNN achieves the highest performance on Precision, Recall, F1, AUC, and AUPR, and prevails other models in a large margin. Therefore, it can be concluded that our proposed ABCD-GGNN representation method achieve a splendid performance on the property prediction for anti-breast cancer candidate drug.

### Characteristics analysis and ablation study

#### Runtime analysis of the compared methods

We conduct the experiments to calculate the mean runtime of ABCD-GGNN and other compared baselines on both ER$\alpha$ value prediction and ADMET property prediction tasks. All experiments are conducted on NVIDIA GeForce RTX 2070. All deep learning methods are set with early stopping. Detailed statistics are shown in Table 4. It can be seen that all deep learning methods take more time compared with traditional machine learning methods. In addition, our proposed ABCD-GGNN takes the most runtime, but the runtime of ABCD-GGNN is still on the same order of magnitude as the other deep learning methods. Since all the prediction tasks are conducted through inductive representation learning, overall, the runtimes of all these methods are acceptable.

**Table 4:** Statistics of the runtime (s) on both ER$\alpha$ value prediction and ADMET property prediction tasks

ER$\alpha$ value prediction		ADMET property prediction	
Method	Runtime	Method	Runtime
Linear Regression	0.0937	SVM	3.7634
Random Forest	3.9162	Bi-LSTM	19.0383
SVM	3.4928	Graph-CNN	62.8520
ABCD-GGNN	73.4433	ABCD-GGNN	76.1681

#### Ablation study of the two representation modules in ABCD-GGNN

To demonstrate the effectiveness of both representation readout: discrete descriptor representation and topological graph representation, we take ablation study on the ABMET prediction task. The results are shown in Table 5. It can be seen that the performance of ABCD-GGNN is better than any single representation readout, which demonstrates that both representation readouts contribute to the final representation and are complementary to each other. Meanwhile, the two representation modules are effectively integrated according to the hyper parameter $\lambda$.

**Table 5:** Ablation study to demonstrate the impact of discrete descriptor representation and topological graph representation for ABCD-GGNN on the ADMET prediction task

Model	Dataset	Precision	Recall	F1
Discrete molecular descriptor representation (w/o)	MN	0.8942	0.8763	0.8823
	HOB	0.8392	0.8550	0.8439
	hERG	0.8547	0.8631	0.8561
	CYP3A4	0.9274	0.9104	0.8967
	Caco-2	0.8584	0.8722	0.8646
Molecular graph representation (w/o)	MN	0.7986	0.7316	0.7471
	HOB	0.8006	0.8348	0.8219
	hERG	0.7618	0.7092	0.7153
	CYP3A4	0.8718	0.8026	0.8193
	Caco-2	0.8162	0.8023	0.8025
ABCD-GGNN	MN	0.9255	0.9613	0.9430
	HOB	0.8637	0.8804	0.8712
	hERG	0.8914	0.8839	0.8842
	CYP3A4	0.9474	0.9163	0.9355
	Caco-2	0.8828	0.8832	0.8829

We run all models 10 times and report the mean test precision, recall, and F1

#### Ablation study of the hyper parameter $\lambda$

**Fig. 3:**
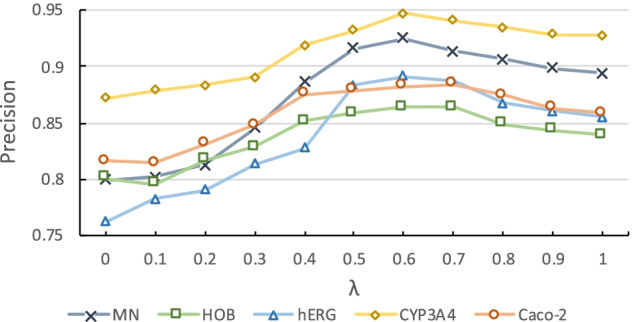
Precision of ABCD-GGNN with a varying $\lambda$ on ADMET prediction tasks

In addition, since our designed hyper parameter $\lambda \in \left( 0,1\right)$ controls the trade-off between the two views of representation, we also conduct the ablation study to seek the optimal value of $\lambda$ for anti-breast cancer candidate drug selection. Figure 3 exhibits the performance of ABCD-GGNN with a varying $\lambda$ on ADMET prediction tasks. $\lambda =1$ means we only utilize the topological molecular graph representation, and $\lambda =1$ means we only utilize the discrete property representation. On all the five property prediction tasks, the precision is consistently higher with larger $\lambda$ value. This can be explained by the high performance of topological molecular graph representation. The model reaches its best when $\lambda =0.6$, performing slightly better than only utilizing topological molecular graph representation.

#### Ablation study of the pooling operation in the readout stage

We designed fusion strategy in the readout stage of ABCD-GGNN, which utilizes both average pooling and max pooling operations to better represent each compound. To demonstrate the effectiveness of the fusion of the two pooling operations, we take the ablation study in terms of the pooling operation selection as is shown in Table 6. It can be seen that our designed fusion strategy does contribute to better representation performance for ADMET prediction tasks. Meanwhile, the average pooling and max pooling operations are complementary to each other.

**Table 6:** Ablation study on the pooling operation in the readout stage of ABCD-GGNN for ADMET prediction

Pooling operation	MN	HOB	hERG	CYP3A4	Caco-2
Average pooling	0.9173	0.8586	0.8840	0.9329	0.8751
Max pooling	0.9086	0.8514	0.8792	0.9245	0.8684
Fusion	0.9255	0.8637	0.8914	0.9474	0.8828

We run all models 10 times and report the mean test precision

#### Ablation study of the interaction step in molecular graph representation

**Fig. 4:**
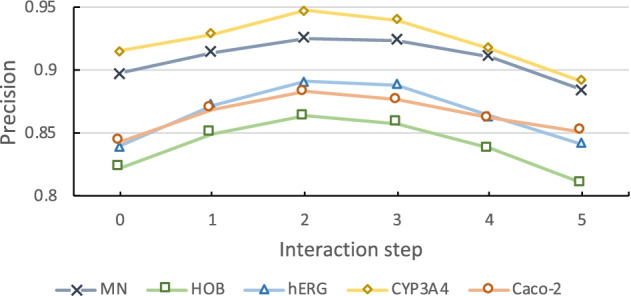
Precision of the molecular graph representation part of ABCD-GGNN with a varying interaction step on ADMET prediction tasks

Interaction step is the key parameter which controls the global information interaction of molecular graph representation. Therefore, we coduct the ablation study to seek the optimal number of interaction step for anti-breast cancer candidate drug selection. Figure 4 presents the performance of molecular graph representation with a varying number of the graph layer on ADMET prediction tasks. The result reveals that with the increment of the layer, a node could receive more information from high-order neighbors and learn its representation more accurately. Nevertheless, the situation reverses with a continuous increment, where a node receives from every node in the graph and becomes over-smooth. On all the five property prediction tasks, the representation method overall reaches its best when interaction step is set as 2.

#### The effect of XGBoost feature selection

**Fig. 5:**
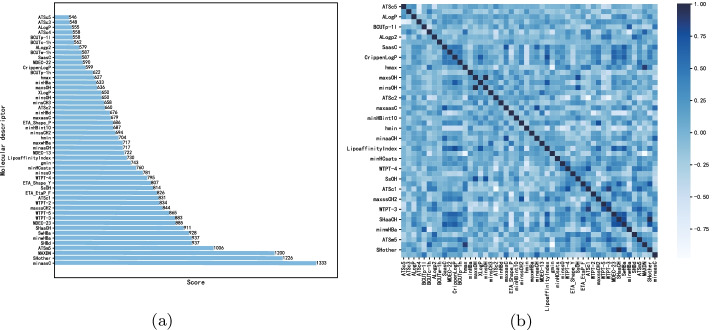
The score list and heatmap of the 50 molecular descriptors selected from the XGBoost in the stage of discrete molecular descriptor representation.** a** Score list,** b** heatmap

In the stage of discrete molecular descriptor representation, a XGBoost is adopted to select the top 50 molecular descriptors, which is intended to reduce the redundancy of the original 729 molecular descriptors. To demonstrate the effectiveness of the XGBoost, we conduct analysis on the 50 molecular descriptors from the XGBoost. The scores of and the heatmap of the selected 50 molecular descriptors are shown in Fig. 5a, b, respectively. It can be seen that the correlation between the selected descriptors are commonly low, which fits our expectation that the 50 molecular descriptors should be in low redundancy.

#### The effect of the ranking operator

**Fig. 6:**
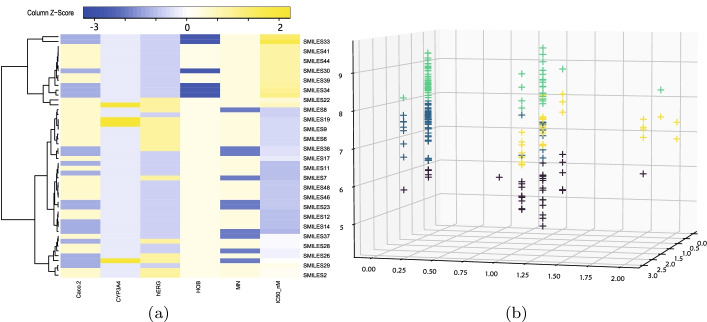
Visualization of the clustering analysis on the results of the ranking operator.** a** Cluster heatmap, the correlation of clustered samples is stronger,** b** k-means clustering analysis

**Fig. 7:**
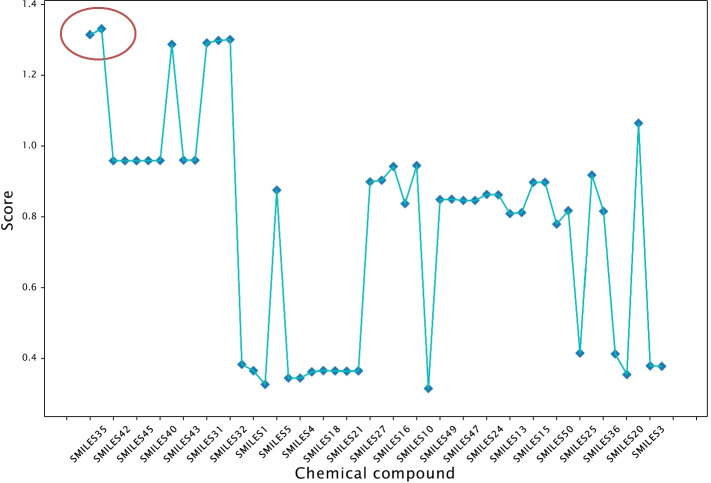
The scoring result of the candidate drugs through the ranking operator

We also conduct result analysis to demonstrate the biological rationality of the ranking operator for the final candidate drug selection. We first comprehensively consider the predicted value of the model’s biological activity value and the classification value of the ADMET property, and perform a cluster analysis on it, as shown in Fig. 6. For example, to analyze the results of cluster analysis, SMILES35 and SMILES33 are classified into one category, and SMILES3 and SMILES2 may also be the same category. Figure 7 shows the quantitative evaluation of the anti-breast cancer ability of the compounds based on the scoring mechanism, where the horizontal axis arranges the compounds in the order of the cluster analysis results in Fig. 6, and the vertical axis represents the scoring of the compounds in this article. It can be seen that the compounds with similar scores are close in the horizontal direction, that is, they are also classified in the same category (with similar properties) in the cluster analysis. For example, two compounds of SMILES35 and SMILES33 belong to the same class and have similar scores. In other words, the ranking operator can make a reasonable quantitative assessment of the compound’s anti-breast cancer ability based on the classification prediction results of the compound.

## Discussion

We evaluated the effectiveness of ABCD-GGNN in predicting ER$\alpha$, and the pharmacokinetic properties and safety of the compounds, by benchmarking on compound dataset containing SMILES and 729 molecular descriptors. In contrast to previous studies, ABCD-GGNN focuses on learning the the structure and substructure characteristics of a candidate drug topologically, and integrating with discrete molecular descriptors to form a more optimal molecular-level representation of feature of a drug.

The experimental results of our method ABCD-GGNN confirm two perspectives to improve the performance of methods for predicting the properties of molecular compounds. From a computational perspective, advanced artificial intelligence methods such as graph neural networks can be utilized to construct a better representation of molecular compound properties based on the structure and substructure of molecules. From a biological perspective, effective integration of structural and substructural features of molecules and other characteristics that reflect the properties of molecules (i.e., molecular descriptors) can better model the characteristic expression of molecular compounds and help researchers understand the biological mechanisms involved. Conclusions above are based on the facts that 1) molecular descriptors can determine the biological activity of compounds as independent variables; 2) graph neural networks enable global feature extraction to further enhance the molecular representation; and 3) as illustrated in Table 5, the ablation experimental results demonstrated that the integration of topological features and discrete descriptor features can further enhance the performance of molecular representation.

If a large number of molecular descriptor classes are available, we suggest using a regression model to evaluate the correlation of descriptors with compound properties and the coupling between descriptors , so as to reduce the redundancy and sparsity of the original molecular descriptors. We analyzed the original 729 molecular descriptors using the XGBoost model, and the results are shown in Table 1, where 50 molecular descriptors with low redundancy status were selected, and they had the highest correlation with the compound properties.

For the selection of anti-breast cancer drugs, we suggest a ranking operator consisting of feature binning and scorecard to select the appropriate anti-breast cancer drugs statistically. Figure 7 shows the quantitative evaluation of the anti-breast cancer ability of the compounds based on the scoring mechanism. Compounds with similar scores can remain similar in the clustering analysis, implying that the ranking operator can comprehensively consider ER$\alpha$, and the pharmacokinetic properties and safety of the compounds, which consists with the biological significance.

In summary, in this paper, we give full consideration to the high correlation between ER$\alpha$ expression and breast cancer, and the significance of ADMET properties of a compound. By employing the ABCD-GGNN representation method, our designed framework can integrate multi-view features of compounds and efficiently select candidate drugs for researchers for further drug discovery. Given the universality and adapatbility of molecular representation methods, it is expectable that such framework, with corresponding modification, can also be utilized for the research on other drug selection and contribute to intelligent administration in the pharmacology community.

## Conclusion

In this paper, we propose the ABCD-GGNN representation method aiming at topologically representing the features of anti-breast cancer candidate drugs and predicting the ER$\alpha$ value and ADMET properties of the organic compounds. With the ranking operator employed, research on the drug selection can be facilitated based on these significant metrics. Our proposed ABCD-GGNN representation method topologically learns both the implicit structure and substructure characteristics of a candidate drug and then deeply integrate them with explicit discrete molecular descriptors to strongly enhance the molecule-level representation. Extensive experiments conducted on our collected anti-breast cancer candidate drug dataset demonstrate that our proposed model outperforms all the other representative methods. Extended analysis also proves the biological rationality of our designed anti-breast cancer candidate drug selection strategy.

## Supplementary Information

**Additional file 1**. Descriptions of components of the feature initialization for the atomic nodes.

## Data Availability

The datasets used and/or analysed during the current study available from the corresponding author on reasonable request.
