# Supplementary material for: Topology-enhanced molecular graph representation for anti-breast cancer drug selection
Source: BMC Bioinformatics. 2022 Sep 19;23:382. doi: 10.1186/s12859-022-04913-6 (PMC9484163; doi:10.1186/s12859-022-04913-6)
Supplement: Supplementary file 1 — Additional file 1. Descriptions of components of the feature initialization for the atomic nodes. [file 12859_2022_4913_MOESM1_ESM.pdf]

Appendix

Supplemetary Table 1 presents the detailed description of the 729 molecular descriptors that are included in the dataset we utilized in this paper.

Table 1 Descriptions of components of the feature initialization for the atomic nodes.

Descriptor Java Class	# of descriptor	Class	Descriptor Java Class	# of descriptor	Class
AcidicGroupCountDescriptor	1	2D	KappaShapeIndicesDescriptor	3	2D
ALOGPDescriptor	3	2D	LargestChainDescriptor	1	2D
APolDescriptor	1	2D	LargestPiSystemDescriptor	1	2D
AromaticAtomsCountDescriptor	1	2D	LongestAliphaticChainDescriptor	1	2D
AromaticBondsCountDescriptor	1	2D	MannholdLogPDescriptor	1	2D
AtomCountDescriptor	14	2D	McGowanVolumeDescriptor	1	2D
AutocorrelationDescriptorCharge	5	2D	MDEDescriptor	19	2D
AutocorrelationDescriptorMass	5	2D	MLFERDescriptor	6	2D
AutocorrelationDescriptorPolarizability	5	2D	PetitjeanNumberDescriptor	1	2D
BasicGroupCountDescriptor	1	2D	RingCountDescriptor	34	2D
BCUTDescriptor	6	2D	RotatableBondsCountDescriptor	1	2D
BondCountDescriptor	10	2D	RuleOfFiveDescriptor	1	2D
BPolDescriptor	1	2D	TPSADescriptor	1	2D
CarbonTypesDescriptor	9	2D	VABCDescriptor	1	2D
ChiChainDescriptor	10	2D	VAdjMaDescriptor	1	2D
ChiClusterDescriptor	8	2D	WeightDescriptor	1	2D
ChiPathClusterDescriptor	6	2D	WeightedPathDescriptor	5	2D
ChiPathDescriptor	16	2D	WienerNumbersDescriptor	2	2D
CrippenDescriptor	2	2D	XLogPDescriptor	1	2D
EccentricConnectivityIndexDescriptor	1	2D	ZagrebIndexDescriptor	1	2D
ElectrotopologicalStateAtomTypeDescriptor	488	2D	CPSADescriptor	29	3D
ExtendedTopochemicalAtomDescriptor	43	2D	GravitationalIndexDescriptor	9	3D
FMFDescriptor	1	2D	LengthOverBreadthDescriptor	2	3D
FragmentComplexityDescriptor	1	2D	MomentOfInertiaDescriptor	7	3D
PaDELHBondAcceptorCountDescriptor	4	2D	PetitjeanShapeIndexDescriptor	2	2D
PaDELHBondDonorCountDescriptor	2	2D	WHIMDescriptor	85	3D
HybridizationRatioDescriptor	1	2D			
